# Glycohypoxia: a hypothesis linking chronic hyperglycemia to functional hypoxia and diabetic complications in type 2 diabetes

**DOI:** 10.4103/mgr.MEDGASRES-D-25-00137

**Published:** 2026-03-14

**Authors:** Maher M. Akl, Amr Ahmed

**Affiliations:** 1 Faculty of Science, Mansoura University, Mansoura, Egypt; 2 Department of Public Health, Riyadh First Health Cluster, Ministry of Health, Riyadh, Saudi Arabia

**Keywords:** GLUT-4 dysregulation, glycohypoxia, HbA1c, hemoglobin glycation, HIF-1α/VEGF signaling, osmotic stress, polyol pathway, RAGE-NF-κB axis, reactive oxygen species, type 2 diabetes

## Abstract

**Facts**

1. HbA1c formation increases hemoglobin oxygen affinity, inducing functional hypoxia despite normal oxygenation.
2. Osmotic stress via aquaporins, Na^+^/K^+^-ATPase, and the polyol pathway disrupts vascular oxygen diffusion.
3. Glycohypoxia integrates glycation, osmotic, and transport defects into a unified hypoxic framework.
4. Chronic hypoxia inducible factor-1α/vascular endothelial growth factor activation drives fibrosis and angiogenesis across diabetic organs.
5. Glucose emerges as both a metabolic and respiratory regulator in diabetes pathophysiology.

**Open Questions**

1. Does chronic hypoxia inducible factor-1α signaling produce adaptive or maladaptive fibrosis in diabetic tissues?
2. Could glycohypoxia promote Warburg-like metabolic shifts linking hyperglycemia to tumorigenesis?
3. Might smoking-related carboxyhemoglobin and HbA1c synergy intensify functional hypoxia?
4. Does glycohypoxia reprogram immune metabolism, explaining chronic inflammation in diabetes?
5. Could small-molecule allosteric effectors (e.g., Efaproxiral) reverse HbA1c-induced oxygen retention?

Given that chronic hyperglycemia in type 2 diabetes induces functional cellular hypoxia by constraining the release of oxygen from hemoglobin, a hypothesis of glycohypoxia was proposed. This hypothesis positions glucose as a novel regulator of respiratory dynamics beyond its metabolic functions. This narrative review aims to unravel the molecular framework of glycohypoxia, reinterpret diabetic complications from a hypoxia-centric perspective, highlight underrecognized hypoxic interconnections, and advocate for innovative hypoxia-targeted therapeutic strategies to transform diabetes management. The glycohypoxia hypothesis illuminates type 2 diabetes as a disorder of impaired oxygen delivery. According to this hypothesis, non-enzymatic glycation of hemoglobin may yield glycated hemoglobin via covalent binding to β-chain N-terminal valine, potentially locking hemoglobin in a high-affinity state, shifting the oxyhemoglobin dissociation curve leftward (the partial pressure of oxygen at which hemoglobin is 50% saturated, P_50_ ≈ 23 mmHg *vs*. 26.8 mmHg), and restricting oxygen unloading, possibly undermining Bohr and Haldane effects. Hyperglycemia may exacerbate this process by driving osmotic stress through glucose transporter-mediated influx, aquaporin-1/3 activation, and sodium-potassium adenosine triphosphatase engagement, resulting in cellular swelling. The polyol pathway, catalyzed by aldose reductase, may convert glucose into sorbitol. This process depletes nicotinamide adenine dinucleotide phosphate and generates reactive oxygen species via nicotinamide adenine dinucleotide phosphate oxidase, thereby impairing glycocalyx integrity and mitochondrial function. Insulin resistance may further compromise glucose transporter type 4 translocation, perpetuating hyperglycemia and limiting adenosine triphosphate synthesis. Overall, these cascades may activate hypoxia-inducible factor-1α, elevate vascular endothelial growth factor and transforming growth factor-β, and promote fibrosis and angiogenesis, contributing to complications, such as retinopathy, neuropathy, nephropathy, cardiomyopathy, and potentially cancer, via Warburg-like metabolic shifts. Therefore, anti-glycation agents (e.g., aminoguanidine), polyol inhibitors (e.g., epalrestat), glucose transporter type 4 agonists (e.g., fisetin), and 2,3-bisphosphoglycerate enhancers can restore oxygen unloading function, improve hyperglycemia, and treat diabetes.

## Introduction

The seamless exchange of oxygen (O_2_) and carbon dioxide (CO_2_) is fundamental to cellular viability, driven by hemoglobin’s finely tuned oxygen-binding affinity. This affinity is modulated by factors such as pH, 2,3-bisphosphoglycerate (2,3-BPG), and the partial pressure of CO_2_, which collectively shape the oxyhemoglobin dissociation curve.^1^,^2^ Glucose, conventionally recognized as the primary substrate for cellular energy production, fuels glycolysis and oxidative phosphorylation to generate adenosine triphosphate (ATP).^3^ However, this traditional perspective limits glucose to a metabolic role, overlooking its potential as a regulator of gas exchange dynamics. In diabetes mellitus, tissues exhibit hallmarks of cellular hypoxia despite adequate blood oxygen levels, a paradox that challenges conventional models of oxygen delivery.^4^ This discrepancy suggests that chronic hyperglycemia impairs oxygen release through molecular mechanisms. One key mechanism involves the non-enzymatic glycation of hemoglobin, forming glycated hemoglobin (HbA1c), which increases hemoglobin’s oxygen affinity, thereby reducing oxygen release to tissues.^5^ Additionally, hyperglycemia induces osmotic stress by activating aquaporins and sodium-potassium ATPase (Na^+^/K^+^-ATPase), disrupting cellular water and ion homeostasis.^6^ These changes may alter erythrocyte membrane fluidity and impair oxygen dissociation. Furthermore, hyperglycemia upregulates hypoxia-inducible factor-1α (HIF-1α), which drives the expression of vascular endothelial growth factor (VEGF), contributing to pathological angiogenesis seen in diabetic complications such as retinopathy.^7^,^8^

Elevated glucose levels also increase the production of reactive oxygen species (ROS), which exacerbate oxidative stress and impair mitochondrial function, further compounding cellular hypoxia.^9^,^10^ Therefore, we proposed the “glycohypoxia” hypothesis, which posits that chronic hyperglycemia precipitates cellular hypoxia by hindering oxygen delivery to tissues, thus redefining glucose as a critical modulator of respiratory physiology. This hypothesis extends to all diabetic complications, including but not limited to retinopathy, neuropathy, nephropathy, cardiomyopathy, and peripheral vascular disease. For instance, diabetic nephropathy may involve hypoxia-driven fibrosis mediated by HIF-1α and transforming growth factor-β (TGF-β),^11^ while neuropathy may stem from impaired oxygen delivery to peripheral nerves, compounded by ROS-induced damage.^12^ Additionally, glucose transporter type 4 (GLUT-4) dysregulation in insulin-resistant states may limit glucose uptake in tissues like skeletal muscle, further exacerbating energy deficits under hypoxic conditions.^13^

This narrative review aims to elucidate the glycohypoxia paradigm, uncover scientific oversights in the current literature, and reinterpret all diabetic complications through the lens of cellular hypoxia. By integrating molecular pathways such as HbA1c-mediated oxygen affinity changes, Na^+^/K^+^-ATPase-driven osmotic stress, HIF-1α/VEGF signaling, ROS production, and GLUT-4 dysregulation, we seek to provide a comprehensive framework for understanding diabetes as a disorder of both metabolism and oxygen delivery. Furthermore, this review proposes innovative research and therapeutic avenues to address this underrecognized phenomenon, including targeting hypoxia-related pathways to mitigate diabetic complications.

## Methods

We employ a structured narrative review to propose the glycohypoxia hypothesis, a conceptual framework suggesting that chronic hyperglycemia in type 2 diabetes contributes to cellular hypoxia and associated complications. A systematic literature search was conducted across PubMed, Scopus, and Web of Science databases, covering publications from 1975 to 2025 to integrate foundational and contemporary insights. Search terms included Medical Subject Headings (MeSH) and free-text keywords such as “glucose dysregulation,” “cellular hypoxia,” “hemoglobin glycation,” “osmotic stress,” “glucose transporter function,” and “type 2 diabetes complications,” combined with Boolean operators (AND/OR) to ensure comprehensive yet targeted retrieval. From an initial pool of 1124 articles, 198 duplicates were removed, leaving 926 for title and abstract screening. Of these, 683 were excluded due to irrelevance, insufficient mechanistic focus, non-English language, limited generalizability (e.g., case reports), or unavailable full texts.

The remaining 243 articles underwent full-text evaluation, with 101 peer-reviewed studies selected based on inclusion criteria emphasizing relevance to glucose-mediated hypoxic mechanisms or diabetic organ dysfunction. Selected studies included clinical trials, *in vitro* experiments, animal models, and biochemical pathway analyses, focusing on molecular processes like HbA1c formation, polyol pathway activity, aquaporin-mediated osmotic changes, and GLUT-4 regulation. Data extraction encompassed study design, target organ system, molecular pathways (e.g., HIF-1α signaling, VEGF expression, receptor for advanced glycation end-products [RAGE]-nuclear factor-κB [NF-κB] activation), and alignment with the glycohypoxia framework. Qualitative synthesis integrated findings across metabolic, hypoxic, and redox biology to construct a cohesive hypothesis, prioritizing conceptual clarity to guide future empirical and therapeutic exploration in type 2 diabetes.

## Conceptual Framework of Glycohypoxia Hypothesis: Physiological Foundations

The delivery of O_2_ from the lungs to peripheral tissues is a cornerstone of cellular metabolism, orchestrated by hemoglobin’s oxygen-carrying capacity within erythrocytes.^14^ Oxygen binds to hemoglobin in pulmonary capillaries, forming oxyhemoglobin, and is released in tissues based on the oxyhemoglobin dissociation curve, which describes hemoglobin’s oxygen-binding affinity as a function of the partial pressure of O_2_.^1^ This process is regulated by allosteric modulators, including pH (via the Bohr effect), 2,3-BPG, and CO_2_, which reduce hemoglobin’s oxygen affinity in metabolically active tissues to facilitate O_2_ unloading.^2^ The Haldane effect enhances gas exchange by promoting CO_2_ release in the lungs as oxyhemoglobin forms.^15^ Molecularly, hemoglobin’s tetrameric structure undergoes conformational changes, with 2,3-BPG binding to deoxyhemoglobin’s β-chains to stabilize the low-affinity tense state, increasing the P_50_ value (the partial pressure of O_2_ at which hemoglobin is 50% saturated).^16^ In diabetes mellitus, tissues exhibit hypoxic signatures despite normal arterial oxygenation, suggesting disruptions in oxygen release that classical modulators (pH, 2,3-BPG, and CO_2_) cannot fully explain.^17^,^18^

Chronic hyperglycemia, a hallmark of diabetes, exerts profound osmotic and biochemical effects that impair oxygen delivery at the tissue level.^19^ Elevated glucose concentrations increase intracellular osmolarity, disrupting water and ion homeostasis through the activation of aquaporins, which facilitate water movement across cell membranes, and Na^+^/K^+^-ATPase, which maintains membrane potential and ion gradients.^20^,^21^ This osmotic stress can lead to cellular swelling, altering the cellular microenvironment and impairing oxygen extraction from surrounding capillaries.^22^ In endothelial cells, for instance, hyperglycemia-induced osmotic shifts disrupt the glycocalyx, a critical barrier that regulates vascular permeability and oxygen diffusion.^23^

Glucose uptake via glucose transporters (GLUTs), particularly glucose transporter type 1 (GLUT-1) and GLUT-4, exacerbates intracellular osmotic pressure by facilitating rapid glucose influx in response to hyperglycemia.^24^ Additionally, the polyol pathway, activated under high glucose conditions, converts glucose to sorbitol via aldose reductase, further increasing osmolarity and generating ROS that damage cellular structures, including mitochondria and cell membranes.^25^ These osmotic and oxidative stresses hinder oxygen diffusion across cell membranes, contributing to the hypoxic phenotype observed in diabetic tissues.^26^ Furthermore, hyperglycemia upregulates HIF-1α, which drives the expression of VEGF, contributing to pathological angiogenesis seen in diabetic complications such as retinopathy, nephropathy, and cardiomyopathy.^27^

Hyperglycemia also impacts oxygen delivery through non-enzymatic glycation of hemoglobin, forming HbA1c, a well-established marker of long-term glycemic control.^28^ Glycation occurs when glucose covalently binds to the N-terminal valine of hemoglobin’s β-chains, altering its structure and function.^29^ HbA1c exhibits increased oxygen affinity, shifting the oxyhemoglobin dissociation curve to the left and reducing the P_50_ value, which impairs oxygen release to tissues. This molecular modification stabilizes the high-affinity relaxed state of hemoglobin, counteracting the effects of 2,3-BPG and pH, and diminishes the efficacy of the Bohr effect. The Haldane effect may also be disrupted, as HbA1c binds CO_2_ less effectively, potentially impairing CO_2_ transport.^1^,^2^ In diabetic patients, elevated HbA1c levels are correlated with a wide range of complications, including microvascular (e.g., retinopathy, neuropathy, nephropathy) and macrovascular (e.g., peripheral vascular disease, coronary artery disease, stroke) complications, all of which are characterized by tissue hypoxia.^28^,^30^

Current literature often overlooks the osmotic and glycation-related effects of glucose in the context of gas exchange, focusing primarily on its metabolic roles. This gap highlights the need to explore glucose’s broader regulatory impact on oxygen delivery.

The glycohypoxia hypothesis suggests that chronic hyperglycemia induces cellular hypoxia by impaired oxygen release and uptake due to osmotic stress, HbA1c-mediated changes in oxygen affinity, and oxidative damage triggered by ROS. These mechanisms contribute to an extensive array of diabetic complications, including retinopathy, neuropathy, nephropathy, cardiomyopathy, peripheral vascular disease, diabetic foot ulcers, and diabetic dermopathy, all of which exhibit hypoxic signatures.

For example, in diabetic nephropathy, hypoxia-driven fibrosis is mediated by HIF-1α and TGF-β, while neuropathy may result from impaired oxygen delivery to peripheral nerves and is exacerbated by ROS-induced axonal damage.^31^,^32^ Additionally, GLUT-4 dysregulation in insulin-resistant states limits glucose uptake in tissues like skeletal muscle, compounding energy deficits under hypoxic conditions.^33^ Diabetic dermopathy, characterized by atrophic skin lesions, may also reflect localized hypoxia due to impaired microvascular oxygen delivery.^34^

The classical physiology of gas exchange, while robust, does not account for the hypoxic signatures in diabetic tissues, where oxygen is abundant in the blood but deficient at the cellular level. The glycohypoxia hypothesis redefines glucose as a modulator of respiratory physiology, integrating osmotic effects, HbA1c-mediated oxygen affinity changes, HIF-1α/VEGF signaling, ROS production, and GLUT-4 dysregulation (**Table 1**). This framework provides a novel lens for interpreting all diabetic complications and underscores the need for research into therapeutic strategies targeting hypoxia-related pathways.

**Table 1:** Core mechanisms of glycohypoxia in type 2 diabetes

Mechanism	Pathophysiological basis	Molecular consequences
HbA1c-induced hypoxia	Glycation of hemoglobin increases oxygen affinity, shifting oxyhemoglobin dissociation curve leftward	Reduced tissue O_2_ unloading, HIF-1α activation, VEGF upregulation
Osmotic stress	Glucose influx via GLUT-1/GLUT-4 activates Na^+^/K^+^-ATPase and aquaporins	Cellular swelling, impaired glycocalyx-mediated O_2_ diffusion
Polyol pathway activation	Aldose reductase converts glucose to sorbitol, consuming NADPH	ROS accumulation, oxidative damage to endothelial barrier
GLUT dysfunction	Hyperglycemia overloads GLUT-1; reduced GLUT-4 activity in insulin resistance	Energy deficits, impaired mitochondrial ATP production

The glycohypoxia hypothesis integrates hemoglobin glycation, osmotic stress, polyol pathway activation, and glucose transporter dysfunction into a unified framework, highlighting how each contributes to tissue hypoxia and oxidative imbalance in type 2 diabetes. ATP: Adenosine triphosphate; GLUT: glucose transporter; HIF-1α: hypoxia-inducible factor-1α; NADPH: nicotinamide adenine dinucleotide phosphate; O_2_: oxygen; ROS: reactive oxygen species; VEGF: vascular endothelial growth factor.

## Glycohypoxia Hypothesis

Glycohypoxia describes a state of cellular hypoxia resulting from glucose-mediated impairments in oxygen release and uptake, distinct from hypoxia due to reduced blood oxygen levels.

In diabetes, persistently elevated glucose disrupts oxygen delivery through three primary molecular pathways: non-enzymatic glycation of hemoglobin, which increases its oxygen affinity; osmotic stress, which alters cellular ion and water dynamics; and defective GLUT function, which exacerbates metabolic and osmotic imbalances.^35^,^36^ These processes converge to create a microenvironment in which oxygen is abundant in the bloodstream but inaccessible to cells, driving tissue dysfunction.

**Figure 1:**
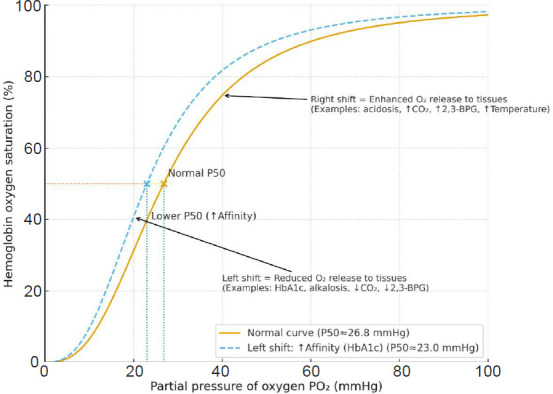
Conceptual oxyhemoglobin dissociation curves illustrating the proposed effect of hemoglobin glycation (HbA1c) on oxygen affinity under chronic hyperglycemia. The solid curve represents normal O_2_–hemoglobin equilibrium under physiological conditions (pH 7.4, 37°C, normal 2,3-BPG; P_50_ ≈ 26.8 mmHg). The dashed curve depicts a hypothetical leftward shift in the presence of HbA1c, reflecting increased O_2_ affinity and impaired tissue unloading (illustrative P_50_ ≈ 23 mmHg). Stable glycation of β-chain N-terminal valine may alter the R↔T conformational equilibrium of hemoglobin, generating “functional hypoxia” despite adequate arterial saturation. Allosteric modifiers such as Efaproxiral (RSR13) produce the opposite effect stabilizing the tense (T) state and enhancing O_2_ release highlighting glycohypoxia as a reversible, targetable biochemical state. Self-generated conceptual model created by the author using GraphPad Prism v10.0 (GraphPad Software, San Diego, CA, USA).

Key molecular players include HbA1c, aquaporins, Na^+^/K^+^-ATPase, and GLUT-4, which collectively mediate the hypoxic phenotype.^37^,^38^,^39^ Glycohypoxia challenges the traditional view of glucose as merely a metabolic substrate, positioning it as a central modulator of respiratory physiology and a driver of diabetic pathology. The molecular underpinnings of glycohypoxia involve interconnected biochemical and biophysical pathways. First, non-enzymatic glycation occurs when glucose covalently binds to the N-terminal valine of hemoglobin’s β-chains, forming HbA1c.^40^ This modification stabilizes hemoglobin’s high-affinity relaxed state, shifting the oxyhemoglobin dissociation curve leftward (decreasing P_50_) and increasing oxygen affinity, thus reducing oxygen release to tissues (**Figure 1**). Glycation also impairs the Bohr effect (pH-dependent oxygen unloading) and Haldane effect (CO_2_-mediated oxygen release), as HbA1c binds CO_2_ less effectively, disrupting gas exchange.^1^,^2^ Second, hyperglycemia elevates intracellular osmolarity via rapid glucose influx through GLUT-1 (constitutive in most cells) and GLUT-4 (insulin-regulated in muscle and adipose tissue).^41^ This triggers aquaporin-1 and aquaporin-3 activation, increasing water influx, and upregulates Na^+^/K^+^-ATPase to restore ion gradients, leading to cellular swelling.^42^,^43^ The polyol pathway further exacerbates osmotic stress, as aldose reductase converts glucose to sorbitol, accumulating osmotically active metabolites and generating ROS via nicotinamide adenine dinucleotide phosphate (NADPH) depletion.^44^ ROS damage the endothelial glycocalyx, impairing oxygen diffusion across vascular barriers.^45^,^46^ Third, insulin resistance impairs GLUT-4 translocation to the plasma membrane, reducing glucose uptake and perpetuating hyperglycemia.^47^

This sustains osmotic stress and limits ATP production via glycolysis and oxidative phosphorylation, compromising oxygen-dependent cellular processes.^48^ These mechanisms glycation, osmotic stress, and GLUT dysfunction synergistically create a hypoxic cellular milieu, as tissues fail to access oxygen despite its vascular availability, forming the molecular basis of glycohypoxia.

In the context of diabetic complications, glycohypoxia provides a unified explanation by linking these pathways to tissue-specific hypoxia. Diabetic retinopathy exemplifies this, where recent retinal oximetry studies demonstrate paradoxically elevated oxygen saturation in retinal arterioles and venules in diabetic patients compared to healthy individuals (101% *vs*. 93% in arterioles; 68% *vs*. 58% in venules), suggesting impaired tissue oxygen extraction rather than hypoxemia.^49^ HbA1c increases oxygen affinity, hindering its release to metabolically active retinal cells, thereby creating a ‘silent hypoxia’ despite blood hyperoxia. This hypoxic stress activates HIF-1α, which upregulates VEGF, promoting pathological neovascularization.^50^

Concurrently, glucose flux through the polyol pathway leads to sorbitol accumulation, increasing osmotic stress and damaging retinal pericytes, further compromising capillary integrity and enhancing leakage.^51^ Additionally, aldose reductase activity in this pathway consumes NADPH, weakening glutathione-mediated antioxidant defenses, thereby allowing ROS accumulation and mitochondrial dysfunction. These events converge on impaired oxidative phosphorylation and ATP deficiency within retinal neurons and supporting cells.^52^

Diabetic neuropathy arises from hypoxic damage to peripheral nerves, where HbA1c-mediated reduced oxygen unloading combines with osmotic stress from sorbitol accumulation in Schwann cells, leading to demyelination and axonal degeneration.^53^ ROS generated via the polyol pathway exacerbate this by oxidizing neuronal lipids and proteins, while GLUT-4 dysfunction in insulin-resistant nerves limits energy production, amplifying hypoxic injury.^54^ HIF-1α upregulation in hypoxic nerves promotes VEGF expression, but chronic activation leads to maladaptive fibrosis rather than repair.^55^

Diabetic nephropathy involves hypoxic glomerular injury, as osmotic stress (via sorbitol and aquaporins) and ROS damage podocytes and mesangial cells, while diminished oxygen delivery impairs tubular reabsorption, accelerating renal decline.^56^ HbA1c contributes by sustaining vascular hyperoxia with tissue hypoxia, activating HIF-1α to drive TGF-β-mediated fibrosis in the glomerulus.^57^ GLUT-1 overexpression in proximal tubules under hyperglycemia further increases osmotic load, promoting epithelial-mesenchymal transition and extracellular matrix accumulation.^58^

Diabetic foot ulcers reflect impaired wound healing due to hypoxic tissue microenvironments, where reduced oxygen release (from high HbA1c) and ROS-mediated endothelial dysfunction suppress angiogenesis and collagen synthesis, critical for tissue repair.^59^ Osmotic swelling in keratinocytes and fibroblasts via aquaporin activation hinders cellular migration, while polyol pathway-induced sorbitol traps water in the extracellular matrix, delaying re-epithelialization.^60^

Diabetic cardiomyopathy features hypoxic myocardial cells, with HbA1c impairing oxygen delivery to cardiomyocytes, leading to HIF-1α-driven metabolic shifts toward glycolysis and reduced contractility.^61^ Osmotic stress via Na^+^/K^+^-ATPase hyperactivity causes calcium dysregulation, while ROS from mitochondrial uncoupling exacerbates apoptosis.^62^,^63^ GLUT-4 translocation defects in insulin-resistant hearts limit substrate availability, compounding ATP deficits under hypoxia.^13^

Peripheral vascular disease manifests as hypoxic limb ischemia, where glycation-stiffened vessels and ROS-induced endothelial nitric oxide synthase uncoupling reduce vasodilation, impairing oxygen perfusion.^64^ Polyol pathway activation in smooth muscle cells promotes sorbitol-mediated osmotic damage and VEGF dysregulation, leading to plaque instability.^65^

Coronary artery disease involves hypoxic cardiac endothelium, with HbA1c reducing oxygen release to atherosclerotic plaques, activating HIF-1α to promote inflammation via NF-κB.^66^ Osmotic stress contributes to plaque rupture through matrix metalloproteinase activation by ROS.^67^

Stroke in diabetes stems from hypoxic cerebral tissue, where GLUT-3 (brain-specific) dysfunction under hyperglycemia sustains osmotic edema, while HbA1c impairs oxygen delivery across the blood-brain barrier, triggering HIF-1α-mediated excitotoxicity.^68^,^69^ ROS from polyol pathway damage neurons, exacerbating infarction.^70^

Diabetic dermopathy presents as hypoxic dermal lesions, with reduced oxygen unloading to skin fibroblasts causing atrophic changes; osmotic stress via aquaporins leads to collagen glycation and ROS-induced pigmentation.^71^,^72^

Gastroparesis results from hypoxic enteric neurons, where HbA1c-mediated oxygen deficits and polyol-induced ROS damage interstitial cells of Cajal, impairing gastric motility.^73^,^74^ GLUT-4 dysfunction in smooth muscle exacerbates energy failure.^75^

Erectile dysfunction arises from hypoxic corpora cavernosa, with endothelial ROS and reduced NO bioavailability from endothelial nitric oxide synthase uncoupling under osmotic stress, compounded by HbA1c-impaired oxygenation.^76^ HIF-1α promotes fibrosis over angiogenesis.^77^

Cognitive impairment in diabetes involves hypoxic hippocampal neurons, where GLUT-1/3 dysregulation causes osmotic brain swelling, HbA1c reduces oxygen supply, and ROS triggers amyloid-beta aggregation via HIF-1α dysregulation.^78^,^79^

These complications share a hypoxic signature, evidenced by elevated HIF-1α and VEGF in diabetic tissues, yet the literature rarely attributes these to glucose’s role in oxygen delivery. Glycohypoxia unifies these pathologies by demonstrating how glycation (altering hemoglobin function), osmotic stress (disrupting cellular homeostasis), and GLUT-4 dysfunction (perpetuating metabolic stress) converge to induce cellular hypoxia, offering a mechanistic explanation for diabetic tissue damage.

Glycohypoxia integrates hemoglobin glycation, osmotic stress, and GLUT dysfunction to explain cellular hypoxia in diabetes, challenging conventional models that focus solely on hyperglycemia’s metabolic effects. By highlighting glucose’s role in impairing oxygen delivery, this hypothesis elucidates the molecular pathogenesis of diabetic complications and identifies critical research gaps. Experimental validation of glycohypoxia, through studies measuring tissue oxygenation in diabetic models or assessing HbA1c’s impact on P_50_, is essential.

Therapeutically, targeting glycation (e.g., anti-glycation agents), osmotic stress (e.g., aldose reductase inhibitors), or GLUT-4 function (e.g., agonists) could mitigate hypoxic damage, offering novel strategies for managing diabetic complications and potentially other hypoxia-related disorders (**Table 2**).

**Table 2:** Traditional *vs*. glycohypoxia perspectives on diabetic complications

Complication	Traditional view (metabolic focus)	Glycohypoxia view (hypoxia from impaired O_2_ delivery)
Retinopathy	Vascular damage from AGEs, sorbitol accumulation, pericyte loss → neovascularization	HbA1c ↑ O_2_ affinity → ↓ retinal O_2_ unloading; osmotic stress & ROS damage pericytes; HIF-1α/VEGF pathological angiogenesis
Neuropathy	Polyol pathway toxicity and oxidative stress causing axonal degeneration	HbA1c-induced hypoxia + sorbitol osmotic swelling in Schwann cells; ROS myelin damage; GLUT-4 dysfunction → ATP failure
Nephropathy	Hyperfiltration, mesangial expansion, TGF-β activation	HbA1c impairs O_2_ unloading; osmotic podocyte injury; ROS/HIF-1α fibrosis; GLUT-1 overload → tubular hypoxia
Foot ulcers	Poor healing from neuropathy + vascular insufficiency	Hypoxic wound bed from HbA1c; polyol ROS ↓ angiogenesis & collagen synthesis; osmotic swelling blocks fibroblast migration
Cardiomyopathy	Lipotoxicity, insulin resistance, fibrosis	Myocardial hypoxia from HbA1c; Na^+^/K^+^-ATPase osmotic stress → Ca²^+^ dysregulation; ROS mitochondrial uncoupling; GLUT-4 ATP deficit
Peripheral vascular disease	Accelerate atherosclerosis from endothelial dysfunction	Limb hypoxia from glycation-stiffened vessels; ROS/eNOS uncoupling → ↓ vasodilation; polyol osmotic SMC injury
Coronary artery disease	Plaque buildup from dyslipidemia + inflammation	Endothelial hypoxia despite vascular hyperoxia; HbA1c ↓ plaque O_2_; ROS/MMP activation; HIF-1α/NF-κB inflammation
Stroke	Vascular damage from hypertension + hyperglycemia	Cerebral hypoxia from HbA1c; GLUT-3 osmotic edema; ROS neuronal injury; HIF-1α excitotoxicity
Dermopathy	Microangiopathy and dermal glycation	Dermal fibroblast hypoxia; aquaporin osmotic collagen injury; ROS-driven pigmentation
Gastroparesis	Autonomic neuropathy from metabolic toxicity	Enteric neuronal hypoxia; polyol ROS injures interstitial cells of Cajal; GLUT-4 ATP failure
Erectile dysfunction	Vascular + neural hyperglycemic damage	Corpora cavernosa hypoxia; ROS/eNOS uncoupling → ↓ NO; HbA1c O_2_ deficit; HIF-1α fibrosis
Cognitive impairment	Insulin resistance → neuroinflammation & amyloidosis	Hippocampal hypoxia from GLUT-1/3 dysregulation; HbA1c ↓ O_2_ delivery; ROS/HIF-1α → amyloid aggregation

The table highlights how impaired oxygen delivery through HbA1c modification, osmotic stress, ROS accumulation, and transporter dysfunction may provide a unifying pathogenic mechanism across multiple organ systems. AGE: Advanced glycation end-product; ATP: adenosine triphosphate; Ca²^+^: calcium ion; eNOS: endothelial nitric oxide synthase; GLUT: glucose transporter; HbA1c: glycated hemoglobin; HIF-1α: hypoxia-inducible factor-1α; MMP: matrix metalloproteinase; Na^+^/K^+^-ATPase: sodium-potassium ATPase; NF-κB: nuclear factor-κB; NO: nitric oxide; O_2_: oxygen; ROS: reactive oxygen species; SMC: smooth muscle cell; TGF-β: transforming growth factor-β; VEGF: vascular endothelial growth factor.

## Clinical Implications and Future Directions of the Glycohypoxia Hypothesis

The glycohypoxia hypothesis suggests that chronic hyperglycemia induces functional hypoxia in diabetic tissues, offering novel clinical and research avenues to address oxygen delivery deficits. By redefining HbA1c as a potential indicator of both glycemic control and tissue hypoxia risk, this framework extends its diagnostic utility. HbA1c, formed by non-enzymatic glucose binding to hemoglobin’s β-chain N-terminal valine, may increase hemoglobin’s oxygen affinity, reducing oxygen release to tissues.^80^ Integrating HbA1c measurements with advanced tissue oxygenation assays, such as near-infrared spectroscopy or oxygen-sensitive probes, could enable early identification of hypoxia-driven risks in diabetic patients.^81^ Additionally, assessing hemoglobin’s allosteric properties, such as P_50_ values, in patients with elevated HbA1c may provide insights into oxygen delivery impairments.^82^ Molecularly, HIF-1α and VEGF expression, induced by hypoxic stress, could serve as biomarkers to track glycohypoxia progression, offering a shift from current metabolic-focused diagnostics.^83^

Therapeutic strategies targeting glycohypoxia’s core mechanisms, hemoglobin glycation, osmotic stress, and GLUT dysfunction, aim to restore oxygen delivery and mitigate diabetic complications. Each intervention addresses specific molecular pathways and complications:

Anti-glycation agents (e.g., aminoguanidine, alagebrium): These compounds may inhibit glucose binding to hemoglobin, reducing HbA1c formation and normalizing oxygen affinity. By preventing advanced glycation end-products (AGEs), which form via non-enzymatic glycation of proteins in vascular matrices, these agents could preserve endothelial integrity and oxygen diffusion.^84^,^85^

Molecularly, AGEs activate RAGE, triggering NF-κB mediated inflammation, which exacerbates vascular stiffness in complications like retinopathy (pericyte loss), nephropathy (glomerular fibrosis), and coronary artery disease (plaque instability).^86^ Anti-glycation agents may thus mitigate these complications by reducing RAGE-NF-κB signaling and enhancing oxygen delivery to hypoxic tissues.^87^ Polyol pathway inhibitors (e.g., epalrestat): These inhibitors target aldose reductase, which converts glucose to sorbitol, reducing osmotic stress and ROS production via NADPH depletion.^88^ Sorbitol accumulation increases intracellular osmolarity, activating aquaporin-1 and aquaporin-3, leading to cellular swelling that impairs oxygen diffusion across membranes.^89^

ROS, generated by NADPH oxidase activation, damage the endothelial glycocalyx, a key regulator of oxygen transport, contributing to complications such as diabetic foot ulcers (impaired angiogenesis), neuropathy (Schwann cell damage), and nephropathy (podocyte injury).^90^ Epalrestat may alleviate these by restoring glycocalyx function and reducing oxidative stress, improving oxygen availability in affected tissues.^91^

GLUT-4 agonists (e.g., fisetin): These insulin-independent enhancers may promote GLUT-4 translocation to the plasma membrane, improving glucose uptake in insulin-resistant tissues like skeletal muscle and adipose tissue.^92^ Hyperglycemia sustains osmotic stress by overloading GLUT-1 in non-insulin-dependent cells, increasing Na^+^/K^+^-ATPase activity and water influx, which disrupts cellular homeostasis.^20^ GLUT-4 agonists could reduce hyperglycemia, alleviating osmotic stress and supporting oxygen-dependent mitochondrial ATP production. This may benefit complications like cardiomyopathy (myocardial ATP deficits), peripheral vascular disease (impaired vasodilation), and cognitive impairment (hippocampal energy failure) by enhancing cellular energy status and oxygen utilization.^93^

2,3-BPG enhancers (e.g., phosphate analogs): These compounds may increase 2,3-BPG levels, stabilizing hemoglobin’s low-affinity tense state to counteract HbA1c’s high oxygen affinity.^80^ Molecularly, 2,3-BPG binds to deoxyhemoglobin’s β-chains, increasing P_50_ and promoting oxygen release. This could enhance oxygen delivery to hypoxic tissues, addressing complications such as stroke (cerebral ischemia), diabetic dermopathy (dermal fibroblast hypoxia), and gastroparesis (enteric neuronal oxygen deficits). By restoring hemoglobin’s allosteric balance, these enhancers may mitigate hypoxia-driven damage across multiple diabetic complications.^94^

Investigating tissue oxygenation in diabetic models using phosphorescence quenching or magnetic resonance imaging could quantify oxygen delivery deficits linked to HbA1c levels.^95^ Studies examining aquaporin-1/3 expression and Na^+^/K^+^-ATPase activity under hyperglycemic conditions may clarify the impact of osmotic stress on oxygen diffusion.^96^,^97^ *in vitro* endothelial models could explore how sorbitol accumulation alters glycocalyx integrity, while animal studies testing GLUT-4 agonists could assess their effects on glucose clearance and tissue oxygenation.^98^ Quantifying HIF-1α and VEGF as hypoxic markers in diabetic tissues may further correlate glycohypoxia with clinical outcomes.^99^ Exploring glycohypoxia’s relevance to non-diabetic hypoxic conditions, such as ischemic heart disease, could broaden its therapeutic impact (**Table 3**). These interdisciplinary approaches aim to address critical gaps in understanding glucose’s role in oxygen delivery, paving the way for hypoxia-centric interventions in diabetes management.

**Table 3:** Therapeutic strategies targeting glycohypoxia and associated diabetic complications

Therapeutic agent	Mechanism	Targeted molecular pathway	Diabetic complications	Evidence level
Anti-glycation agents (e.g., aminoguanidine, alagebrium)	Inhibit HbA1c formation and AGEs, normalize oxygen affinity, reduce vascular inflammation	Block glucose binding to hemoglobin; inhibit RAGE-NF-κB signaling	Retinopathy, nephropathy, coronary artery disease	Preclinical
Polyol pathway inhibitors (e.g., epalrestat)	Reduce sorbitol accumulation and ROS, alleviate osmotic stress, protect endothelial glycocalyx	Inhibit aldose reductase; restores NADPH and glycocalyx function	Neuropathy, diabetic foot ulcers, nephropathy	Early clinical
GLUT-4 agonists (e.g., fisetin)	Enhance GLUT-4 translocation, improve glucose uptake, reduce osmotic stress	Promote insulin-independent glucose clearance; supports mitochondrial ATP production	Cardiomyopathy, peripheral vascular disease, cognitive impairment	Preclinical
2,3-BPG enhancers (e.g., phosphate analogs)	Increase 2,3-BPG levels, stabilize hemoglobin’s tense state, promote oxygen release	Enhance P_50_ via allosteric modulation of hemoglobin	Stroke, diabetic dermopathy, gastroparesis	Preclinical

In addition to specifying molecular pathways and associated diabetic complications, the table incorporates an "Evidence Level" column to distinguish between preclinical findings and early clinical applications. This refinement highlights both the translational potential and current limitations of these approaches, framing glycohypoxia as a central and targetable mechanism in diabetic complications. 2,3-BPG: 2,3-Bisphosphoglycerate; AGE: advanced glycation end-product; ATP: adenosine triphosphate; GLUT-4: glucose transporter type 4; HbA1c: glycated hemoglobin; NADPH: nicotinamide adenine dinucleotide phosphate; NF-κB: nuclear factor-κB; RAGE: receptor for advanced glycation end-product; ROS: reactive oxygen species.

## Discussion

The glycohypoxia hypothesis proposes a transformative shift in understanding diabetes mellitus, suggesting that chronic hyperglycemia contributes to functional hypoxia by impairing cellular oxygen utilization, thus redefining glucose’s role beyond its traditional metabolic function. This paradigm challenges the prevailing focus on hyperglycemia’s metabolic and vascular consequences, positing that oxygen delivery deficits represent a unifying mechanism underlying diabetic pathology. By integrating molecular, physiological, and clinical perspectives, glycohypoxia offers a novel framework to address critical gaps in current research, with far-reaching implications for diagnostics, therapeutic innovation, and interdisciplinary collaboration, potentially extending to hypoxia-related disorders beyond diabetes, such as ischemic heart disease or cancer.

The hypothesis’s strength lies in its ability to unify disparate aspects of diabetic pathology under the lens of functional hypoxia. Unlike conventional models that attribute tissue damage to metabolic toxicity or vascular insufficiency, glycohypoxia emphasizes the interplay between glucose and oxygen delivery, highlighting overlooked mechanisms that may drive tissue dysfunction.

This perspective necessitates a reevaluation of diagnostic approaches, which currently prioritize glycemic markers over hypoxic indicators. For instance, integrating advanced techniques like near-infrared spectroscopy to assess tissue oxygenation or monitoring molecular hypoxic signals, such as HIF-1α and VEGF expression, could enable earlier detection of at-risk patients. Molecularly, HIF-1α activation under hypoxic stress induces VEGF transcription, promoting angiogenesis that may be maladaptive in diabetic tissues, contributing to pathological remodeling. Such insights suggest that hypoxia-centric biomarkers could complement existing metabolic assays, enhancing risk stratification and personalizing patient care.

Therapeutically, the hypothesis inspires innovative strategies targeting glucose-mediated hypoxic pathways. Interventions that modulate hemoglobin’s oxygen affinity, such as compounds enhancing 2,3-BPG levels, could restore oxygen release to tissues by stabilizing hemoglobin’s low-affinity tense state, addressing hypoxic deficits.

Similarly, agents targeting the RAGE-NF-κB axis may reduce inflammation-driven vascular dysfunction, which exacerbates hypoxia by impairing endothelial barrier integrity.^96^ Inhibiting aldose reductase activity could mitigate osmotic stress and ROS production, preserving the endothelial glycocalyx a critical regulator of oxygen diffusion and supporting cellular homeostasis.^97^

Enhancing GLUT-4 translocation in insulin-resistant tissues may stabilize energy metabolism by improving glucose clearance, thereby supporting mitochondrial ATP production under hypoxic conditions. These approaches, grounded in the hypothesis’s molecular insights, contrast with current therapies focused solely on glycemic control, offering a complementary hypoxia-centric paradigm to improve clinical outcomes.^98^

The glycohypoxia hypothesis also highlights significant research gaps that warrant urgent investigation. The lack of comprehensive studies quantifying oxygen delivery deficits in diabetic tissues represents a critical barrier to validating this framework. Experimental models using techniques like phosphorescence quenching or magnetic resonance imaging could elucidate the relationship between glycemic markers and tissue oxygenation, providing mechanistic clarity. *in vitro* studies by observing endothelial cell responses to hyperglycemic conditions may reveal how glucose alters oxygen transport dynamics, particularly through glycocalyx remodeling or aquaporin-mediated water flux. Clinical trials evaluating hypoxia-targeted therapies, such as those modulating hemoglobin allostery or osmotic stress, are essential to translate these insights into practice.

Moreover, exploring the hypothesis’s relevance to non-diabetic hypoxic disorders offers a broader impact. For example, in cancer, glucose-driven hypoxic microenvironments activate HIF-1α, promoting tumor angiogenesis and metastasis, suggesting shared molecular pathways with diabetes.^99^ Similarly, in ischemic heart disease, glucose dysregulation may exacerbate myocardial hypoxia, indicating potential therapeutic overlap.^100^ These interdisciplinary connections underscore the hypothesis’s potential to reshape metabolic and hypoxic disease research.

Despite its promise, the glycohypoxia hypothesis faces challenges that require rigorous validation. The absence of direct causal evidence linking glucose-mediated mechanisms to tissue hypoxia necessitates longitudinal studies in diverse diabetic populations. Variability in hypoxic responses across tissues due to differences in vascularity, metabolic demand, or GLUT expression complicates clinical translation. For instance, tissues with high GLUT-1 expression, like the brain, may experience distinct hypoxic dynamics compared to insulin-dependent tissues like skeletal muscle.^101^ Addressing these complexities requires integrated approaches combining molecular biology, physiology, and clinical research. Additionally, the hypothesis’s reliance on emerging biomarkers, such as HIF-1α or VEGF, demands standardized assays to ensure reproducibility across studies. These challenges, while significant, present opportunities to advance metabolic medicine by fostering collaboration across disciplines.

The broader impact of glycohypoxia lies in its ability to bridge metabolism and gas exchange, challenging researchers to rethink the pathophysiology of diabetes and related disorders.

By positioning glucose as a modulator of oxygen delivery, it encourages a holistic approach to clinical guidelines, integrating hypoxic mechanisms into diagnostic and therapeutic frameworks. This paradigm could influence fields like oncology, where hypoxic microenvironments drive tumor progression, or cardiology, where oxygen deficits underpin ischemic pathology. While limitations, such as the need for robust experimental evidence, remain, the glycohypoxia hypothesis provides a compelling foundation for future discoveries, urging scientists and clinicians to explore this uncharted territory and redefine the boundaries of metabolic disease research.

## Limitations and Considerations

The glycohypoxia hypothesis, while proposing a novel framework for understanding cellular hypoxia in type 2 diabetes, requires careful consideration of several challenges to ensure robust validation. These challenges are presented proactively to guide future research and strengthen the hypothesis’s applicability, focusing on molecular and experimental rigor without implying definitive conclusions.

One challenge is establishing whether HbA1c, formed by glucose binding to hemoglobin’s β-chain N-terminal valine, consistently reduces the P_50_ value *in vivo* in humans, thereby impairing oxygen release. Current understanding suggests HbA1c may increase hemoglobin’s oxygen affinity, potentially shifting the oxyhemoglobin dissociation curve leftward, but compensatory mechanisms like elevated 2,3-BPG levels could mask this effect. To address this, studies could measure P_50_ in erythrocytes from patients stratified by HbA1c levels (e.g., < 6.5%, 6.5–8%, > 8%), using standardized tonometry and co-oximetry to control for pH, temperature, and anemia. Quantifying 2,3-BPG via enzymatic assays would clarify its compensatory role, ensuring precise attribution to HbA1c’s molecular impact on oxygen-binding kinetics.

Another consideration is isolating HbA1c’s effect on P_50_ from confounding factors like pH, CO_2_, 2,3-BPG, and temperature, which modulate hemoglobin’s oxygen affinity. Diabetic states often involve acidosis or altered CO_2_ levels, complicating P_50_ measurements. To overcome this, experiments could employ controlled conditions (pH 7.4, 37°C, fixed partial pressure of carbon dioxide buffers) to standardize measurements, excluding patients with anemia (hemoglobin < 12 g/dL) or acid-base imbalances.

This approach, leveraging respiratory physiology protocols, would ensure HbA1c’s specific contribution is accurately assessed, minimizing interference from multifactorial influences.

The osmotic stress mechanism, driven by hyperglycemia-induced glucose influx via GLUT-1 and GLUT-4, activating aquaporin-1/3 and Na^+^/K^+^-ATPase, also requires mechanistic validation. While cellular swelling may impair oxygen diffusion by altering the endothelial glycocalyx, direct evidence is limited. *In vitro* studies using endothelial monolayers under high-glucose conditions (e.g., 25 mM *vs*. 5 mM) could measure oxygen flux with Clark electrodes or phosphorescence quenching, alongside assessing glycocalyx integrity via lectin staining and aquaporin expression through quantitative polymerase chain reaction or Western blot. These experiments would elucidate how osmotic stress disrupts oxygen transport at the molecular level.

Finally, the scope of glycohypoxia as an explanatory model for all diabetic complications warrants scrutiny, as it may complement rather than fully replace established pathways like AGEs or RAGE-NF-κB-mediated inflammation. For instance, glycohypoxia may intersect with AGE-driven vascular damage by amplifying HIF-1α and VEGF signaling, adding a hypoxic dimension to traditional mechanisms. Comparative studies in animal models could evaluate glycohypoxia’s contribution relative to inflammatory or metabolic pathways, identifying synergistic interactions to refine its explanatory power (**Table 4**).

**Table 4:** Challenges, responses, and proposed experimental approaches

Challenge	Response	Proposed experimental approach
Establishing HbA1c’s consistent reduction of P_50_ *in vivo*	Recognize potential compensatory effects of 2,3-BPG	Measure P_50_ in HbA1c-stratified cohorts with tonometry/co-oximetry and enzymatic assays
Isolating HbA1c’s effect from confounders (pH, CO_2_, 2,3-BPG, temperature)	Require controlled physiological conditions	Standardize assays at pH 7.4, 37°C, fixed PCO_2_; exclude anemia/acid-base disorders
Validating osmotic stress’s impairment of oxygen diffusion	Limited direct evidence; propose mechanistic validation	High-glucose endothelial monolayers to assess O_2_ flux, glycocalyx integrity, aquaporin expression
Determining glycohypoxia’s scope *vs*. other mechanisms	Frame as complementary to AGE/RAGE and inflammatory pathways	Comparative animal studies measuring tissue PO_2_, HIF-1α/VEGF *vs*. AGEs/NF-κB

The table emphasizes the need for precise measurements of hemoglobin oxygen affinity, osmotic effects, and comparative studies, ensuring robust evaluation of the hypothesis’s explanatory power. 2,3-BPG: 2,3-Bisphosphoglycerate; AGE: advanced glycation end-product; CO_2_: carbon dioxide; HbA1c: glycated hemoglobin; NF-κB: nuclear factor-κB; PO_2_: partial pressure of oxygen; RAGE: receptor for advanced glycation end-product; VEGF: vascular endothelial growth factor.

By proactively addressing these challenges, the glycohypoxia hypothesis can be rigorously tested, fostering a deeper understanding of its molecular and clinical implications in type 2 diabetes. This narrative review lacks quantitative meta-analysis due to heterogeneous studies, and relies on selective synthesis, potentially introducing bias. Future systematic reviews could address these gaps.

## Conclusion

The glycohypoxia hypothesis illuminates type 2 diabetes as a disorder of impaired oxygen delivery, where glucose-mediated pathways, such as hemoglobin glycation (HbA1c formation stabilizing R state), osmotic stress (aquaporin/Na^+^/K^+^-ATPase activation and polyol-driven sorbitol/ROS accumulation), and GLUT-4 dysfunction (reduced translocation limiting ATP), orchestrate cellular hypoxia across complications. Retinopathy involves HIF-1α/VEGF-induced neovascularization; neuropathy, ROS-mediated demyelination; nephropathy, TGF-β fibrosis; cardiomyopathy, mitochondrial uncoupling; peripheral vascular disease, endothelial nitric oxide synthase uncoupling; and cognitive impairment, amyloid aggregation. These insights challenge metabolic-centric paradigms and advocate the use of hypoxia biomarkers (HIF-1α and VEGF) and diagnostic tools like tissue oximetry. Therapeutic prospects encompass anti-glycation agents that can mitigate RAGE-NF-κB inflammation, polyol inhibitors that can preserve glycocalyx, GLUT-4 agonists that can bolster energy metabolism, and 2,3-BPG enhancers that can restore oxygen release. Despite validation needs via imaging and trials, glycohypoxia fosters interdisciplinary advances, potentially extending to ischemic or neoplastic hypoxia, redefining diabetes management through integrated molecular and physiological strategies.

## Data Availability

*Not applicable.*
